# The experience of intolerance of uncertainty for parents of young people with a restrictive eating disorder

**DOI:** 10.1007/s40519-021-01256-8

**Published:** 2021-07-22

**Authors:** Anna Konstantellou, Lot Sternheim, Lucy Hale, Mima Simic, Ivan Eisler

**Affiliations:** 1 Maudsley Centre for Child and Adolescent Eating Disorders, South London and Maudsley NHS Foundation Trust, London, UK; 2 Department of Clinical Psychology, Utrecht University, Utrecht, The Netherlands; 3 Department of Psychology, University of Surrey, Guildford, UK

**Keywords:** Anorexia nervosa, Intolerance of uncertainty, Parents, Focus groups, Qualitative research

## Abstract

**Purpose:**

This study aimed to explore how parents of young people with a restrictive eating disorder (ED) experience and manage uncertainty.

**Methods:**

Seventeen parents of young people with a restrictive ED were recruited from multi-family therapy groups run within a specialised ED clinic. Five focus groups were conducted asking parents about their experience of uncertainty both prior and after the onset of their child’s illness.

**Results:**

Data were analysed using interpretative phenomenological analysis which yielded seven superordinate themes. (1) Anorexia nervosa and uncertainty, (2) Positive and negative experiences of uncertainty (3), Helpful and unhelpful ways of coping with uncertainty, (4) Parent’s self-efficacy and uncertainty (5), Needs of parents, (6) Parents’ perceptions of intolerance of uncertainty in their children and (7) Impact of uncertainty on family life.

**Conclusion:**

Parents caring for young people with a restrictive ED exhibit a strong intolerance of uncertainty, particularly in relation to their child’s illness. This ‘negative uncertainty’ was thought to reduce their confidence as parents in how they managed their child’s ED. Targeting high levels of intolerance of uncertainty in parents caring for young people with an ED could be beneficial for supporting parents when faced with their child’s illness, increasing parental self-efficacy, decreasing accommodating behaviours and ultimately contributing to improved treatment outcomes.

**Level of evidence:**

Level V: Opinions of authorities, based on descriptive studies, narrative reviews, clinical experience, or reports of expert committees.

## Introduction

Intolerance of uncertainty (IU) has been defined as a personality trait that is future orientated and results in a cognitive bias regarding uncertainty and its implications [1]. It suggests that individuals who are “intolerant of uncertainty” have a negative reaction on a cognitive, emotional and behavioural level when confronted with an uncertain situation [2]. IU was initially developed within the anxiety literature and later on became part of the IU model for generalised anxiety disorder (GAD) [3]. According to this model, IU is implicated in the development and maintenance of worry, the main symptom of GAD, by propelling “what if…” questions, in both adults [4] and adolescents [5].

Since then, IU has been considered as a shared cognitive factor across GAD, depression and obsessive compulsive disorder [6] and a causal factor for anxiety related symptoms [7]. Evidence is growing supporting IU is a trans-diagnostic maintaining factor for both anxiety and depression and an indicator of psychological distress in general [8]. A recent study found changes in IU to result in an increase of DSM-V incidence of psychological disorders [9].

Within the eating disorder (ED) literature research has been limited. A review of 21 studies identified heightened levels of IU in individuals with anorexia nervosa (AN) when compared to healthy controls [10]. Indeed, elevated levels of IU have been identified in both a non-clinical sample of individuals with ED difficulties [11] and in clinical samples of individuals with AN [12–16]. In particular, IU seems relevant to restrictive type EDs, with people with AN generally reporting higher levels of IU compared to people with bulimia nervosa [10]. Qualitative findings from focus groups of adults with AN further support and extend findings from quantitative studies revealing that uncertainty is viewed as a negative experience to be avoided at all costs that results in increased levels of anxiety, stress and sense of loss of control [16].

Even less is known of the role of IU in adolescents with an ED. Results from a recent qualitative study show that adolescents during treatment for AN, experience difficulties managing uncertainty and use certain ED-related behaviours as coping strategies when faced with uncertainty [17]. Furthermore, positive correlations have been identified between high levels of IU and AN psychopathology in young people compared to healthy controls [12, 18]. Clinically, patients with AN could benefit from interventions targeting IU which subsequently could reduce comorbid levels of anxiety symptoms and the need for control considered as key to understanding AN and should be addressed in treatment [19]. Results from a pilot study on the benefits of targeting IU in adolescents with AN provide initial support [20].

In the treatment of AN in children and adolescents, families are central with ED focused family therapy (FT-ED) recommended as the first line of treatment [21]. Within this treatment approach, a key premise is that parents play an important role in their child’s recovery. Unfortunately, quite often carers of those with AN feel out of their depth and struggle to provide the required supporting role.

Typical of being a carer for someone with an ED is uncertainty, around the disorder itself, but also regarding treatment (progress) and general thoughts about the future (“will this ED go away”). It is thus to be expected that those particularly intolerant for uncertainty will struggle when caring for someone with an ED. However, the concept of IU has not previously been investigated in parents of those with an ED.

Research into carer experiences of uncertainty has mainly focused on investigating uncertainty in carers of individuals with a physical illness as opposed to a psychological disorders and dates back to the work of Mishel in the early 1980s. According to Mishel’s model [22], uncertainty plays a prominent role in how parents perceive and manage their child’s physical illness. The more parents experience uncertainty, ambiguity, lack of clarity and unpredictability concerning their child’s illness, the greater their distress, which impacts how they manage their child’s illness altogether [22, 23].

Concordance of cognitive features, such as IU, between parents and children has been demonstrated. For instance, mothers with high IU are more likely to have children with high IU [24]. A more recent study also detected levels of IU in college students comparable to their mothers [25]. It has been shown that GAD runs in families [26, 27] and thus underlying vulnerability factors of GAD, such as IU, are also likely to be intergenerational within families [28]. We might then expect parents of young people with AN to also show high levels of IU given the high levels of IU detected in young people with AN [12, 17, 18]. A negative perception and response to uncertainty as a family could ultimately result in the family remaining caught up in unhelpful patterns of responding to the ED. Trying new ways of dealing with the disorder and accepting uncertainty are key in facilitating treatment progress and thus high levels of IU within a family could compromise such progress.

Given the severity of the illness and uncertain nature of recovery, exploring parental experiences of uncertainty could therefore be a fruitful line of enquiry. As yet, there is no research examining the concept of uncertainty in family members of individuals suffering from an ED. The purpose of the present study was to explore IU in parents of young people with a restrictive ED. Our study focused on parents of young people with a restrictive presentation since according to previous research, such individuals are more likely to have high levels of IU compared to those with bulimia nervosa [10]. Furthermore, parents were recruited from specific multi-family therapy groups run for families caring for young people suffering from a restrictive ED. Finally, homogeneity of groups was considered important. Including parents of young people with different ED presentations could have compromised the generation of more focused themes and our understanding of what uncertainty is like for parents caring for a young person with a restrictive ED. Therefore, the present study did not consider other EDs.

## Materials and methods

### Participants

Purposive sampling was used to recruit parents from three consecutive Multi-Family Therapy (MFT) groups for AN [29, 30] at the Maudsley Centre for Child and Adolescent Eating Disorders. All seventeen parents that were approached gave written consent and were recruited into the study. Table 1 shows demographic characteristics of parents in each group, as well as, information on the young people they were caring for. No same-gender families were present in any of the MFT groups.

**Table 1:** Demographic characteristics of parents and young person caring for in each focus group

Focus Group	N	Parent	Ethnicity	Mean age (*SD)*	Diagnosis YP (N)	Mean age YP (*SD*)	Illness Duration (months)
FG1	3	Mother	WB	47.33(5.51)	AN-R (5)	14.80 (1.78)	8–27
FG2	4	Father	WB	53.50(11.27)	»	»	»
FG3	4	Father	3 WB; 1 Asian	50.40 (6.31)	AN-R (2)/EDNOS-R (3)	16.20 (1.30)	8–16
FG4	3	Mother	2 WB; WO	48.00 (5.21)	»	»	»
FG5	3	Mother	2 WB; 1 MR	47.33 (2.10)	AN-R (3)	14.33 (2.08)	8–24

FG1& FG2 were mothers and fathers from the same families and the same was true of FG3 & FG4

*YP* Young People, *SD* Standard Deviation, *WB* White British, *MR* Mixed Race, *WO* White Other, *AN-R* Anorexia Nervosa Restrictive type, *EDNOS* Eating Disorders Not Otherwise Specified

### Design and topic guide

A focus group design was chosen for the study which would allow shared meanings on a specific topic to emerge [31]. The topic guide used for the focus groups was the same as the one used in two previous studies on uncertainty in individuals with AN [16, 17] with an additional question tailored to parents, examining whether their experiences of uncertainty were different at a previous stage of their life (see “Appendix”).

### Procedure

Five focus groups were run, three with mothers (FG1, FG4 & FG5) and two with fathers (FG2 & FG3). FG1 & FG2 were mothers and fathers from the same families and the same was true of FG3 & FG4. While FG5 were mothers from a separate group. Having separate focus groups for mothers and fathers increased homogeneity, allowed to explore potential gender differences and avoided possible confounding dynamics between members of the same family in one group.

All focus groups were moderated either by the first author or a team clinician while the third or fourth author helped facilitate group discussions and made notes on group dynamics. Due to running focus groups at parallel sessions, it was not possible to have the same moderator and facilitator for all groups. All groups lasted approximately 45 min.

### Analysis

Focus group data were transcribed verbatim by the first author and cross-checked by the third author. Interpretative Phenomenological Analysis (IPA) [32] was chosen as the most appropriate method of analysis. IPA is concerned with in depth explorations of how people experience certain phenomena, how they make sense of them and what meaning they attach to them [33, 34]. IPA acknowledges that total access to a person’s subjective world is not feasible and an element of interpretation by the researcher will always be involved [33]. The transcripts were analysed using a bottom up process of analysis for each transcript, whereby subordinate themes were identified and then grouped into broader super-ordinate themes [33].

### Group dynamics

Discussions across five groups were characterised as supportive, warm and friendly. This positive environment was likely due to the fact that parents in each group already knew one another from attending MFT and could identify with each other’s’ experiences and the struggles they faced. Moderator perceptions and notes from facilitators revealed a few differences between the mothers’ groups and the fathers’ groups. Specifically, fathers often talked in more ‘mechanical terms’ and opted for a problem-solving approach when dealing with uncertainty associated with their child’s illness. This was not observed in mothers’ groups who used more emotive language and were more vocal about their worries. No major differences were identified in presence or absence of IU-related themes between mothers’ and fathers’ groups.

## Results

Seven superordinate themes and nine subordinate themes were identified. Table 2 illustrates presence and absence of themes for each of the groups separately.

**Table 2:** Presence and absence of super-ordinate and subordinate themes in each of the five focus groups

Superordinate themes	Subordinate themes	FG1-Mothers	FG2-Fathers	FG3-Fathers	FG4-Mothers	FG5-Mothers
1. AN and uncertainty	No subordinate themes	√	√	√	√	√
2. Experiences of uncertainty and AN	2.1. Negative experiences of uncertainty	√	√	√	√	√
	2.2. Positive experiences of uncertainty	√	√	√	√	√
3. Coping with uncertainty	3.1. Helpful coping 3.2. Difficulties in coping	√ √	√ X	√ √	√ √	√ X
4. Parents’ roles and abilities	No subordinate themes	√	√	√	√	√
5. Parents’ needs	5.1. Need to take care of young person and understand the illness 5.2. Need for professional advice and guidance	√ √ √	√ √ √	√ X X	√ X X	√ √ √
	5.3. Need for social support and understanding	√	X	X	√	X
	5.4. Need to resume normal life					
6. Young people and uncertainty	No subordinate themes	√	X	√	√	√
7. Impact of uncertainty on family life	7.1. Impact of anorexia nervosa related uncertainty on the family	X	√	√	√	√
	7.2. Impact of anorexia nervosa related uncertainty on family relationships	√	√	X	√	X

*AN* Anorexia nervosa,*√* present in group, *X* not present

### AN and uncertainty

Parents across all five groups viewed uncertainty as an integral part of their child’s ED and the source of intolerable amounts of distress. AN was perceived as a very serious, life-threatening illness, and thought different from physical illnesses, where you could follow a doctor’s prescription. The uncertain course of the illness also contributed to great amounts of concern.

‘…the most challenging thing is the uncertainty of the anorexia it’s so painful.’ (FG1, Father 2).

‘….this [illness] with [name of young person], is uncertainty I’ve never experienced before, I can’t be laid back about it, I panic about it whereas with my job it’s irrelevant but this is something very close to our hearts, totally different type of uncertainty.’ (FG3, Father 8).

‘…anorexia is so uncertain the recovery is so uncertain there is no guarantee that full recovery will take place...’ (FG5, Mother 16).

### Experiences of uncertainty and AN

Parents could identify uncertain situations which were experienced both positively and negatively.

#### Negative experiences of uncertainty

All parents were able to talk about negative experiences of uncertainty much more often than positive experiences. Some negative experiences that were mentioned related to their own lives, for example, employment but more often related to negative experience in relation to their child’s illness. This type of uncertainty was one they had not experienced before and was perceived as quite different to uncertainty in everyday life, which was much more manageable. Some parents talked about the characteristics that made uncertainty negative, such as the threat of a worst-case scenario happening, having little control over the situation and not knowing the outcome of an uncertain situation. When faced with negative uncertainty, particularly in the context of the AN, parents expressed strong negative reactions, on both cognitive and emotional levels.

‘I think the unpleasantness [of uncertainty] is not feeling you have any control... but also what is the worst that can happen.’ (FG3, Father 10).

‘I would say out of all the uncertainties I had in my life, which have been a huge amount, this [uncertainty linked with AN] is probably the most severe...’ (FG5, Mother 15).

‘Terrifying... it is fear a really strong kind of like gut fear I think its coz it is your child coz you would do anything…’ (FG1, Mother 2).

#### Positive experiences of uncertainty

A number of parents across all groups described uncertainty as positive and an essential part of life, without which life would be boring. Uncertainty, and more specifically tolerating uncertainty, was further seen as a positive character trait since it prepares one for life, which simply is unpredictable.

‘…if everything was absolutely certain I think I would be really bored to death…So I think uncertainty can actually be very positive and energizing…’ (FG1, Mother 2).

‘I like a bit of uncertainty...’ (FG4, Mother 13).

‘It [tolerating uncertainty] is character building in the long term’ (FG5, Mother 15).

### Coping with uncertainty

Parents discussed both helpful and unhelpful coping strategies when faced with uncertainty.

#### Helpful coping

When prompted to discuss how one copes with uncertainty parents in all groups identified various helpful strategies, including accepting what one can and cannot control over a situation, looking at possible positive outcomes of the uncertain situation, or taking time out from the situation.

‘...in a work situation how I tend to deal with uncertainty is what can I control, what can I not control, if I can’t control it how can I influence it… and how actually can I get what I need...’ (FG3, P10).

‘... time out is a very important thing...’ (FG5, Mother 17).

#### Difficulties in coping

Parents in FG1, FG3, & FG4 discussed that they found coping with uncertainty difficult and how certain things were unhelpful. Techniques that were thought useful in dealing with uncertainty in life in general were felt inappropriate for the uncertainty that related to their child’s illness. This theme appeared to capture both difficulties surrounding managing uncertainty and difficulties with coping with their child’s illness the two of which were very much interlinked. Indeed, some parents discussed struggling with uncertainty through their child’s illness and others more directly explained that they did not know how to cope with uncertainty and would get into a panic state.

‘... and then you get more and more exhausted and less and less able to cope so it’s like a vicious cycle...’ (FG1, Mother 3).

‘So its weighing up those risks somehow is far more difficult than my future job sort of thing, you are far more emotionally involved...’ (FG3, Father 10).

‘I don’t really [manage uncertainty] I get in a panic...’ (FG4, Mother 14).

### Parents’ roles and abilities

This theme was prominent across all groups. Parents talked about how they felt de-skilled when faced with the uncertainty around their child’s illness and how this affected their confidence as parents and their decision-making skills. They felt their parental abilities were constantly under scrutiny, which they believed was more so than if their child would not have been ill. They discussed feeling frustrated that they could not rely on things that worked in the past to guide their judgment and decisions as their child’s responses were very unpredictable.

‘I think uncertainty can be imprisoning, it makes you feel indecisive...’ (FG2, Father 11).

‘...one of the most difficult things is the fact that it [AN] changes, you are constantly on shifting sand...’ (FG2, Father 6).

‘...I think it is very scary, makes you very lonely... uncertainty takes away your confidence...’ (FG4, Mother 14).

### Parents’ needs

A number of needs were discussed by most of the parents. This theme addressed the concept of IU indirectly by capturing for example the frustration parents experienced with the uncertainty that surrounded their understanding of their child’s illness, and a need for consistency and certainty from professionals as well as, support from friends.

#### Need to take care of young person and understand the illness

Parents in all five groups expressed how the most important thing in their lives was their children’s health and to see them recover. Everything else, such as holidays, their child’s education, and their own needs, came second. AN was experienced as a paradox and difficult to come to grips with, resulting in a strong need to understand its nature.

‘I think its coz it is your child coz you would do anything, it is such a deep instinct to protect your child…’ (FG1, Mother 2).

‘I mean I do find it quite difficult to understand the actual problem because it is just completely alien to me. I can’t see how someone would not want to eat ...’ (FG3, Father 9).

‘…I feel bad to think of my own needs…’ (FG5, Mother 16)

#### Need for professional advice and guidance

A strong need for certainty and clarity from professionals when discussing treatment plans and managing such a serious and unpredictable illness was particularly expressed by parents in FG1 but also in FG2 and FG5.

‘I would just love for somebody to say to me ‘your daughter has anorexia, just do this, or give her this pill and she will be fine’, that’d be fantastic…’ (FG1, Mother 2).

‘Consistency of opinions you don’t expect professionals to have all the answers but some consistency...’ (FG5, Mother 16).

#### Need for social support and understanding

Parents in FG1, FG2 and FG5 expressed the need for more emotional support and understanding from friends and family. Although friends and family were a source of comfort, parents felt that they were not understood as much as they would have needed and that meeting other people going through a similar situation was considered helpful.

‘I suppose talking to other people helps, coming into a forum like this helps where people can identify.’ (FG1, Mother 1).

‘Yeah I think you know having support if people understood a bit more would make a huge difference’ (FG5, Mother 15).

#### Need to resume normal life

A need for normality in one’s life was mentioned in FG1 and FG4. Amongst all the uncertainty, stress, concern and unpredictable nature of the AN holding on to something normal or doing something normal, such as going on a holiday, was something parents longed for.

‘So I do kind of quite like it when I do something normal’ (FG1, Mother 3).

‘...I would really like to go back to my work...’ (FG4, Mother 14).

### Young people and uncertainty

Across the majority of groups, parents talked about young people being distressed in the face of uncertainty and change, as well as, a having a strong need to know the future, need for structure and control. AN was thought to function as a means of dealing with a fear of uncertainty and a lack of control. Some parents expressed how this IU seen in their children was frustrating for them.

‘… I think [young person name] gets quite frightened about change and uncertainty and she gets consumed in the fear and that is what I can’t bear I wish she could let a bit more in of uncertainty without getting so much frightened about it and I think she has to try to lock everything down in a sense and the eating in a way is to do that…’ (FG1, Mother 3).

‘I think she can’t let go of the anorexia because of the uncertainty that awaits her beyond anorexia…’ (FG4, Mother 12).

‘Thinking about other things that have happened last week and the week before that sort of tipped her over the edge where all things that were quite uncertain...’ (FG5, Mother 15).

### Impact of uncertainty on family life

Parents discussed a number of ways in which uncertainty surrounding the AN affected their family life and relationships.

#### Impact of AN-related uncertainty on the family

Parents in the majority of groups, apart from FG1, identified a number of ways in which the presence of AN and the uncertainty associated with it, affected family life. For instance, the family as a whole was thought to have changed to accommodate the presence of the illness. This included allowing space for things not to go according to plan, avoiding any uncertain situations and becoming very rigid as a family. Planning and taking decisions regarding the future became much more challenging than usual.

‘...so basically, you begin avoiding situations that have an uncertain outcome that is what we do so our lives become very very predictable and very very safe...’ (FG4, Mother 12).

‘I kind of almost feel that I have surrendered myself to uncertainty, the days where nice happy family and how quickly it changed from that and since that time it has been very painful and nothing has been certain...’ (FG5, Mother 16).

#### Impact of AN-related uncertainty on family relationships

A number of relationship dynamics were discussed by parents in FG1, FG2 and FG4 as being disrupted due to the elevated levels of uncertainty accompanied with AN. For example, in FG4, one parent felt that her child was using her illness as a way to stay close to her and increase her need for certainty. Parents further discussed how their lives were at the mercy of their children’s illness and accompanied uncertainty.

‘...which particular hoop they are going to have you jumping through which particular week...’ (FG2, Mother 5).

‘...you know she feels she has to be by my side and the way to do that is to be an anorexic you know and it’s so so draining...’ (FG4, Mother 14).

## Discussion

The study aimed to explore parents’ subjective experience of uncertainty whilst caring for a young person with a restrictive ED. Parents highlighted how caring for someone with AN is associated with high levels of IU, which was perceived as negative and accompanied by heightened feelings of distress and anxiety. The excessive amounts of uncertainty associated with AN were further thought to contribute to the disruption of family life, negatively affecting parents’ confidence in their parenting skills. Parents expressed their need for normality, understanding from friends and family and clear guidance from professionals. A frustration with their children’s need for certainty and fear of uncertainty was also discussed.

One dominant theme involved parents expressing a strong dislike towards uncertainty in relation to their child’s illness that was described as ‘negative uncertainty’ they had never experienced before and was often accompanied by feelings of anxiety, stress, frustration, fear, and despair. Although most parents agreed that uncertainty could have positive sides and evoke feelings of excitement, this was never the case with uncertainty related to their child’s illness. Parents also perceived their children as being fearful of uncertainty themselves and very rigid as a response. This supports previous studies highlighting high levels of IU in both adults [12–14] and young people with AN [12, 17, 18].

The seriousness of the illness and the uncertainty associated with it were perceived as negatively affecting the family as a whole. In particular, parents discussed how negative outcomes were feared in the face of uncertainty and a need for stability and certainty was sought. Such findings echo current treatment models that suggest families taking care of an adolescent suffering from AN become ‘re-organised’ around the illness and immobilised in the ‘here and now’, with their own strengths and resources being overlooked [35]. Present findings highlight how IU may lead parents to engage in accommodating and enabling behaviours that inadvertently support rather than challenge their child’s ED. For instance, parents discussed avoiding situations that had an uncertain outcome, modifying their routines to cater for the ED and overall being at the mercy of the illness with family life becoming very rigid. Not all parents accommodate to the illness to the same degree. However, identifying factors, such as IU, that may be underlying accommodating and enabling of AN symptoms could prove useful since these maladaptive strategies are known to affect treatment outcomes for young people with AN [36].

Results further revealed how parents who described themselves as able to cope with levels of uncertainty in normal life struggled dealing with the uncertainty associated with their child’s illness. This was described as negatively affecting their confidence as parents. Parental control and self-efficacy are recognised as key components that make up family therapy and have been found as strong predictors of treatment outcome for adolescent with AN above AN related behaviours [37, 38]. In fact, the higher the levels of parents’ self-efficacy, the better the treatment outcome for young people undergoing family therapy for an ED [38]. Clinically then attending to parents’ IU, particularly concerning their child’s illness could prove beneficial in increasing parental self-efficacy and optimising AN treatment outcomes.

The present study has several strengths and limitations. First, the study had a good sample size of 17 parents, with either three or four participants in each group. Usually, small sample sizes are favoured when carrying out IPA [33]. Both mothers and fathers were included in the study but were seen in separate focus groups, which allowed for more homogeneous samples to exist in each focus group. A number of group dynamics were observed as different between mothers and fathers but no major IU topic-related differences were detected indicating themes were relevant for both mothers and fathers. Second, the focus group schedule was tailored to explore uncertainty currently and at previous stages of their life prior to the presence of their child’s illness, allowing for a more global understanding of parental experiences of uncertainty. Credibility of findings was established through reflexivity, which involved being aware of the potential conscious or unconscious biases of the researcher (first author) during the data collection and analysis process. Specifically, prior to any analysis, initial reflections by moderator and facilitator of the group were noted alongside moderator and facilitator backgrounds and potential impact on analysis and group dynamics. For example, reflections were noted on how groups dynamics, given the age difference between parents and researchers and previous background in running focus groups on the topic of IU could have influenced the analysis process. Triangulation, at each major step of the analysis was further validated by the third author.

In terms of limitations, the results were not cross-validated with parents themselves which did not enable to check whether the interpretations reached by the researchers were accurately representing parents’ views. Furthermore, caution needs to be taken regarding the transferability of the findings. This is a qualitative study involving parents who were in a specific therapeutic setting and happened to all be from two-gender parent families. Having parents from the same family taking part in the focus groups albeit in different groups could have introduced intrafamilial nested effects. Taking care of the same young person undergoing treatment for an ED and living in the same household could result in a shared understanding and response to uncertainty. However, looking at similarities/differences between mothers and fathers from same and different households was beyond the scope of the present study. Furthermore, some of the themes generated in the focus groups could be a reflection of the conceptual framework of the MFT approach. Finally, not all groups were run by the same moderator and facilitator, which may have influenced how parents responded.

## Conclusion

In conclusion, findings from the present study emphasise the importance of IU as a factor associated with parents’ distress, lack of confidence in one’s parental abilities and engagement in less adaptive strategies, such as enabling and accommodating behaviours. IU is a promising area for future research with clinical relevance that could reduce parental distress, increase well-being and potentially help improve treatment outcomes for young people with a restrictive ED.

## What is already known on this subject?

Research into uncertainty in parents has focused on those caring for someone with a physical illness. IU has not been previously investigated in parents of those suffering from a restrictive ED despite parents playing a key role in their child’s recovery and faced with such an uncertain and serious illness.

## What does this study add?

Findings add to existing knowledge of contributing factors to parental distress suggesting IU is a relevant concept for parents of those suffering from a restrictive ED. Themes highlight possible mechanisms IU could be reducing parental self-efficacy and increasing accommodating behaviours.

## Topic Guide

We would like to understand more about what your experience of what it feels like when things are uncertain in your life. In life, we all come up against uncertainty some of the time and we can have all sorts of reactions to this. We want to learn more about what leads you to feel uncertain and what the experience of uncertainty is like for you in the various life domains in which it can crop up.

- Can you think of an uncertain situation?
- What makes this situation uncertain?
- What features of the situation were most troubling?
- Can you tell us a little more about what that experience of uncertainty (or ‘not knowing’) is like for you?
- Can you tell us more about the thoughts and feelings that come up when you are uncertain?
- What makes these thoughts and feelings worse?
- What helps at these times?
- Are there other ways you try and cope?
- Are there good sides of feeling uncertain?
- Who experiences uncertainty? Everyone? Some people? Special groups?
- Can you tell us now whether the experience of uncertainty was different in any way at a different time in your life?
